# Restoration of Podocyte Structure and Improvement of Chronic Renal Disease in Transgenic Mice Overexpressing Renin

**DOI:** 10.1371/journal.pone.0006721

**Published:** 2009-08-21

**Authors:** Anne-Cécile Huby, Maria-Pia Rastaldi, Kathleen Caron, Oliver Smithies, Jean-Claude Dussaule, Christos Chatziantoniou

**Affiliations:** 1 INSERM UMR 702, Paris, France; 2 Fondazione IRCCS Ospedale Maggiore Policlinico & Fondazione D'Amico per la Ricerca sulle Malattie Renali, Milano, Italy; 3 Department of Molecular and Cell Physiology, University of North Carolina, Chapel Hill, North Carolina, United States of America; 4 Department of Pathology and Laboratory Medicine, University of North Carolina, Chapel Hill, North Carolina, United States of America; 5 Université Pierre et Marie Curie-Paris VI, UMR S 702, Paris, France; 6 AP-HP, Hôpital St-Antoine, Department of Physiology, Paris, France; L' Istituto di Biomedicina ed Immunologia Molecolare, Consiglio Nazionale delle Ricerche, Italy

## Abstract

**Background:**

Proteinuria is a major marker of the decline of renal function and an important risk factor of coronary heart disease. Elevated proteinuria is associated to the disruption of slit-diaphragm and loss of podocyte foot processes, structural alterations that are considered irreversible. The objective of the present study was to investigate whether proteinuria can be reversed and to identify the structural modifications and the gene/protein regulation associated to this reversal.

**Methodology/Principal Findings:**

We used a novel transgenic strain of mouse (RenTg) that overexpresses renin at a constant high level. At the age of 12-month, RenTg mice showed established lesions typical of chronic renal disease such as peri-vascular and periglomerular inflammation, glomerular ischemia, glomerulosclerosis, mesangial expansion and tubular dilation. Ultrastructural analysis indicated abnormal heterogeneity of basement membrane thickness and disappearance of podocyte foot processes. These structural alterations were accompanied by decreased expressions of proteins specific of podocyte (nephrin, podocin), or tubular epithelial cell (E-cadherin and megalin) integrity. In addition, since TGFβ is considered the major pro-fibrotic agent in renal disease and since exogenous administration of BMP7 is reported to antagonize the TGFβ-induced phenotype changes in kidney, we have screened the expressions of several genes belonging in the TGFβ/BMP superfamily. We found that the endogenous inhibitors of BMPs such as noggin and Usag-1 were several-fold activated inhibiting the action of BMPs and thus reinforcing the deleterious action of TGFβ.Treatment with an AT1 receptor antagonist, at dose that did not decrease arterial pressure, gradually reduced albuminuria. This decrease was accompanied by re-expression of podocin, nephrin, E-cadherin and megalin, and reappearance of podocyte foot processes. In addition, expressions of noggin and Usag-1 were markedly decreased, permitting thus activation of the beneficial action of BMPs.

**Conclusions/Significance:**

These findings show that proteinuria and alterations in the expression of proteins involved in the integrity and function of glomerular and renal epithelial phenotype are reversible events when the local action of angiotensin II is blocked, and provide hope that chronic renal disease can be efficiently treated.

## Introduction

Numerous clinical studies defined proteinuria as a major marker of the decline of renal function. In addition, several studies demonstrated that proteinuria is an important risk factor of coronary heart disease and suggested to incorporate proteinuria into the assessment of an individual's cardiovascular risk [1]. Proteinuria occurs when the structure of podocytes, peculiar ramified glomerular cells, is destroyed by disruption of the slit-diaphragm and loss of foot processes. It is generally believed that this structural alteration is the crucial step characterizing the irreversibility of chronic kidney disease.

Our group has investigated over the last years the mechanisms involved in the development of renal fibrosis in order to identify targets for therapy [2]–[8] and was among the first groups to report that regression of renal disease was feasible following therapy with angiotensin II receptor antagonists, at least in experimental models of hypertensive nephropathy [9]–[10]. These results were independently confirmed and extended to additional experimental models of nephropathy by other investigators [11]–[14]. However, a major criticism about reversibility of chronic kidney disease in rodents was that the disease was induced in young animals not suffering for a long period from a chronic disease like hypertension or diabetes (as it usually occurs in humans) and that therapy was induced before reaching huge proteinuria or an important destruction of podocyte structure.

To address these issues in the present study, we used a novel model [15]–[16] of hypertension-induced renal disease mimicking closer the kinetics and the physiopathological characteristics of human nephroangiosclerosis. We found that these mice are hypertensive and display albuminuria as early as 2–3 month old, and that these pathological features are accentuated with age and are accompanied by functional and structural alterations typical of chronic renal disease including loss of podocyte foot processes. We decided to start treatment with an AT1 receptor antagonist when the animals reached the age of 12-months, thus, to treat aged animals that have been proteinuric for a long period of their life-span. We found that treatment with an AT1 receptor antagonist induced reappearance of foot processes and of proteins characterizing normal slit diaphragms, and re-established the normal phenotype of tubular epithelial cells. These changes were accompanied by a shift in the equilibrium between pro and anti-fibrotic members of the TGFβ/BMP superfamily.

This study, by showing that long lasting proteinuria, disorganisation of podocyte structure and phenotype change of tubular epithelium can be reversed supports the notion that chronic renal disease can be efficiently treated.

## Results

### RenTg mice as a model to study hypertension-induced disease

RenTg mice were generated and described previously [15]. A major advantage of this transgenic strain is that renin is produced ectopically (in the liver, and thus its release is independent of renal perfusion pressure and electrolyte concentration at the macula densa) at a genetically controlled rate allowing a “standardized” increase of endogenous synthesis of angiotensin II [15]. RenTg mice display elevated systolic blood pressure as early as 2 month old (146±8 compared to 110±4 mm Hg for age matched wild type, p<0.01); at the age of 3 month they show slightly increased albuminuria (18.3±3.5 vs 1.5±0.1 g/mol creat, p<0.01). At this early age, renal morphology as revealed by Masson's trichrome appears to be normal (data not shown).

### RenTg mice develop chronic kidney disease

**Figure 1:**
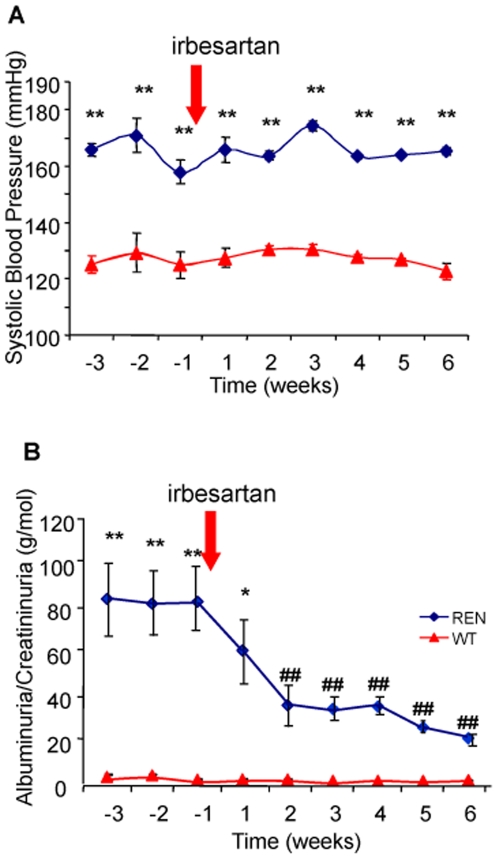
Effect of AT1 receptor antagonism on blood pressure and albuminuria. Systolic blood pressure remained unchanged whereas albuminuria decreased to almost normal levels in 12 month old RenTg mice during administration of the AT1 receptor antagonist irbesartan; in triangles are shown the values for age-matched wild type controls. Values are mean±SEM; n = 5, 9 and 13 for wild type and RenTg mice before and after irbesartan, respectively; * P<0,05 or ** P<0,01 vs WT; ^##^ P<0,001 vs RenTg.

**Figure 2:**
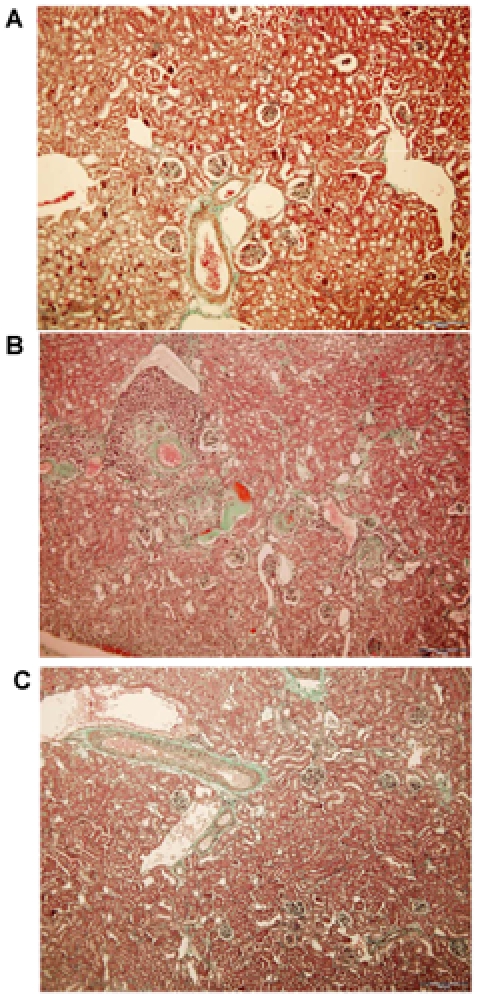
Improvement of renal histology following AT1 receptor antagonism. Representative example of renal cortical histology revealed by Mason's trichrome in 12 month old wild type (A) and RenTg mice before (B) and after (C) 6 weeks of irbesartan administration. Note the substantial improvement of the renal histology following therapy with the AT1 receptor antagonist. Bar = 200 µm.

**Figure 3:**
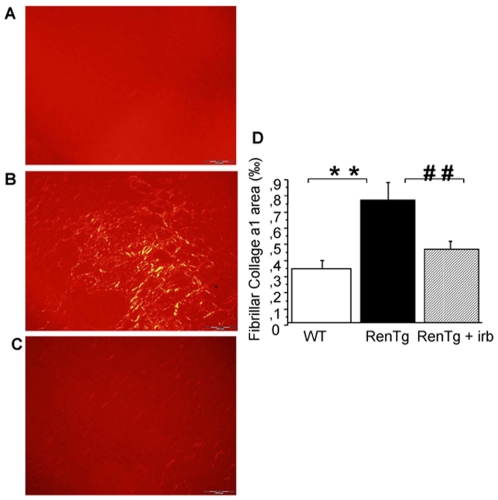
Decrease of renal fibrosis during AT1 receptor antagonism. Representative example of fibrillar collagen content in the renal interstitium revealed by Sirius red staining and polarized light in wild type (A) and RenTg mice before (B) and after (C) irbesartan treatment. Quantification by morphometric analysis is shown on the lower panel (D). Fibrillar collagen content was decreased to normal levels in RenTg mice after irbesartan administration. Values are are mean±SEM; n = 5, 9 and 13 for wild type and RenTg mice before and after irbesartan, respectively; ** P<0,01 vs WT; ^##^ P<0,01 vs RenTg. Bar = 200 µm.

**Figure 4:**
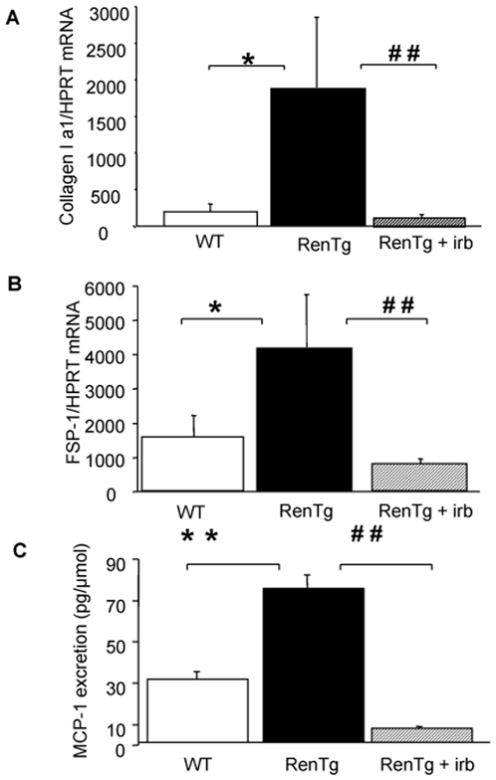
AT1 receptor antagonism led to decreased expression of pro-fibrotic and pro-inflammatory factors in the kidney. Quantitative RT-PCR analysis of the expression of fibrogenic factors such as collagen type I chain a1 (A) and FSP-1 (B) in the renal cortex and urinary excretion of the pro-inflammatory factor MCP-1 (C) in wild type and RenTg mice before and after irbesartan treatment. Note the substantial decrease of these factors following AT1 receptor antagonism. Values are mean±SEM; n = 5, 9 and 13 for wild type and RenTg mice before and after irbesartan, respectively; * P<0,05 or ** P<0,01 vs WT; ^##^ P<0,01 vs RenTg.

Arterial blood pressure and urinary excretion of albumin progressively increased with age to reach at 12 months values highly elevated compared to age matched wild type controls (p<0.001, Fig. 1). Histological examination of kidneys of 12-month old RenTg revealed the presence of well-established lesions in all renal compartments such as peri-vascular and periglomerular inflammation, fibrinoid-like deposits within renal vessels, glomerular ischemia, glomerulosclerosis, mesangial expansion and tubular dilation (Fig. 2B). Fibrillar collagen assessed by red Sirius staining, examined with polarized light and measured by morphometric analysis, increased two-fold in RenTg mice (p<0.01, Fig. 3B). Accordingly, the expression of agents promoting fibrogenesis, such as collagen type I and FSP-1, or indicating renal inflammation, such as MCP-1, were several-fold increased compared to age-matched normotensive controls (p<0.05, p<0.05 and p<0.01, respectively, Fig. 4).

### Ultrastructural modifications in kidneys of RenTg mice

**Figure 5:**
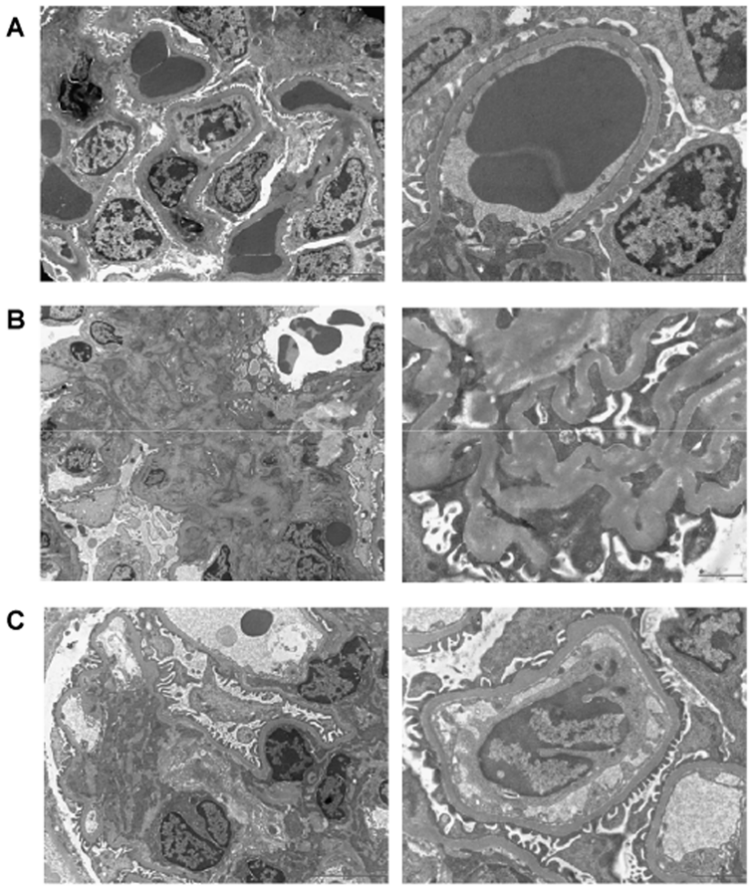
Restoration of podocyte structure following AT1 receptor antagonism. Representative examples of ultrastructural analysis performed by electron microscopy in wild type (A) and RenTg mice before (B) and after (C) irbesartan treatment. The structural alterations of podocytes in RenTg mice such as loss of podocyte foot processes and abnormal thickness of the basal membrane (B) were substantially improved after antagonism of the AT1 receptor (C). Bar = 2 µm.

To assess that proteinuria is associated with important modifications of podocyte structure, ultrastructural analysis by electron microscopy was performed in the renal cortex of 12 month-old RenTg mice and their wild type controls. Figure 5B shows a representative example of the lesions found in all samples of RenTg mice. In particular, podocyte foot processes lost their normal shape, and displayed numerous areas of effacement. The glomerular basement membrane displayed abnormal thickness. Additionally, mesangial expansion was evident.

**Figure 6:**
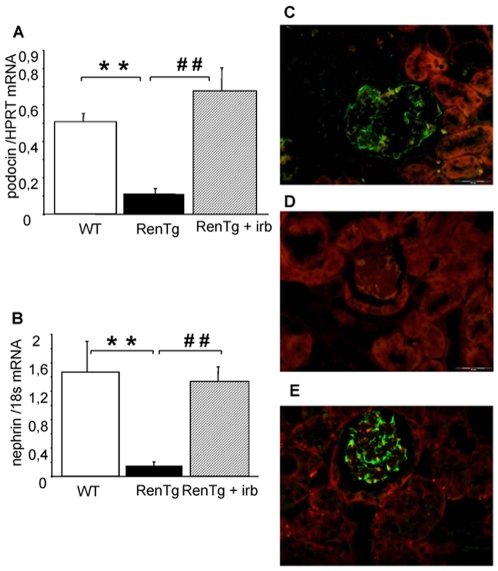
Reestablishment of normal podocyte phenotype after AT1 receptor antagonism. Quantitative RT-PCR analysis of the expression of genes involved in the podocyte structure such as podocin (A) and nephrin (B) in the renal cortex of wild type and RenTg mice before and after irbesartan treatment, and representative examples of podocin expression in glomeruli of wild type (C) and RenTg mice before (D) and after (E) irbesartan treatment. The expressions of podocin and nephrin in the RenTg mice returned to control values following irbesartan administration. Bar = 50 µm. Values are mean±SEM; n = 5, 9 and 13 for wild type and RenTg mice before and after irbesartan, respectively; ** P<0,01 vs WT; ^##^ P<0,001 vs RenTg.

**Figure 7:**
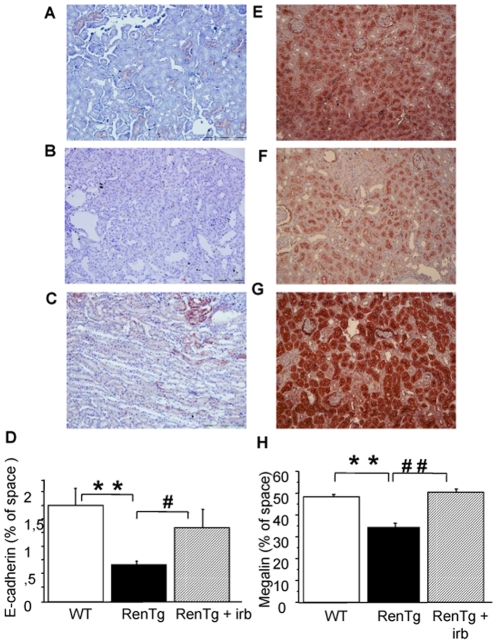
Therapy with AT1 receptor antagonist led to reappearance of proteins characterizing normal function of proximal tubules. Representative examples of the expression of proteins typical of the structure and function of proximal tubules such as E-cadherin and megalin. E-cadherin immunostaining in wild type (A) and RenTg mice before (B) and after (C) irbesartan treatment. Quantification by morphometric analysis is presented in D. Megalin expression in wild type (E) and RenTg mice before (F) and after (G) irbesartan treatment. Quantification by morphometric analysis is presented in H. Note that the blunted expression of both proteins in RenTg mice was increased to normal levels following treatment with the AT1 receptor antagonist. Bar = 200 µm. Values are mean±SEM; n = 5, 9 and 13 for wild type and RenTg mice before and after irbesartan, respectively; ** P<0,01 vs WT; ^#^ P<0,05 or ^##^ P<0,001 vs RenTg.

To provide a cellular assessment of the above-described modifications, we investigated the expression of two proteins essential for the normal structure of podocytes: nephrin and podocin. As shown in Figure 6, nephrin and podocin mRNA expressions were 10- and 5-fold decreased in RenTg, compared to age-matched wild type controls. In addition, two tubular proteins, E-cadherin (an index of normal epithelial phenotype) and megalin (involved in protein reabsorption) were also significantly decreased (Fig. 7B and 7F).

### Restoration of podocyte structure, tubular epithelial phenotype and decrease of proteinuria following AT1 receptor antagonism

To test whether long lasting proteinuria and the related structural lesions can be reversed, 12 month-old RenTg mice were treated with irbesartan for a period of 6 weeks. In parallel, irbesartan was also administered in a group of aged matched wild type controls (n = 5). At the dose used, irbesartan did not affect blood pressure (170±3 mm Hg, Fig. 1A). Despite the lack of anti-hypertensive effect, albuminuria progressively decreased in irbesartan-treated mice (p<0.01 vs RenTg, Fig. 1B). Irbesartan administration did not affect blood pressure nor albuminuria in wild type mice (127±3 mm Hg and 0.9±0.2 g/mol creat, respectively)

This irbesartan-induced decrease in albuminuria in RenTg mice was accompanied by improvement in renal cortical morphology (Fig. 2C), regression of fibrillar interstitial collagen content (Fig. 3C) and normalization of the expression of collagen type I, FSP-1 and MCP-1 (Fig. 4). Irbesartan administration had no effect on renal morphology, fibrogenesis or inflammation in wild type mice (data not shown).

Most important, though mesangial expansion and GBM thickness were not influenced by treatment, podocyte structure recovered almost completely. (Fig. 5C).

The re-establishment of podocyte structure following AT1 receptor antagonism in RenTg mice was associated to the induction of nephrin and podocin expressions at normal levels (Fig. 6) and to re-appearance of E-cadherin and megalin in the proximal tubule (Fig. 7C and 7G). Irbesartan administration did not change expression of the above-mentioned proteins in wild type mice.

### Changes in the TGFβ/BMP equilibrium during progression and reversal of Chronic Kidney Disease in RenTg mice

**Figure 8:**
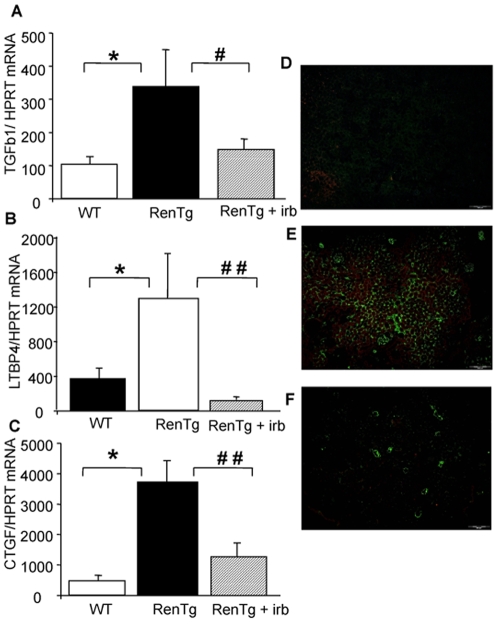
Expression of pro-fibrotic genes of the TGFβ family is substantially decreased following AT1 receptor antagonism. Quantitative RT-PCR analysis of the expression of the pro-fibrotic genes TGFβ (A), CTGF (B) and LTBP4 (C) in the renal cortex of wild type and RenTg mice before and after irbesartan treatment, and TGFβ immunostaining in wild type (D) and RenTg mice before (E) and after (G) irbesartan treatment. Note that the increased expression of pro-fibrotic genes in RenTg mice was decreased to normal levels following treatment with the AT1 receptor antagonist. Values are mean±SEM; n = 5, 9 and 13 for wild type and RenTg mice before and after irbesartan, respectively; * P<0,05 vs WT; ^#^ P<0,05 or ^##^ P<0,001 vs RenTg. Bar = 200 µm.

**Figure 9:**
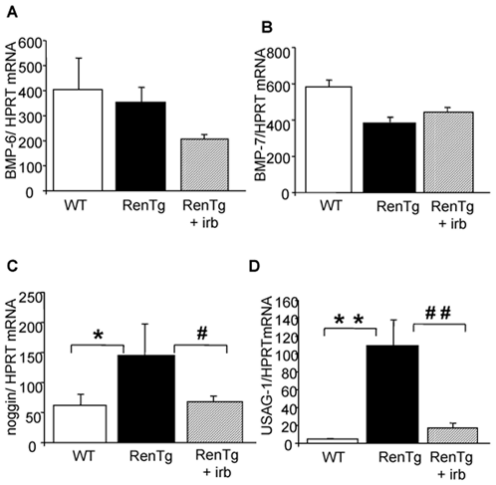
Expression of BMPs and their endogenous inhibitors during progression and reversal of chronic kidney disease. Quantitative RT-PCR analysis of BMP6 (A) and BMP7 (B) and their endogenous inhibitors noggin (C) and USAG-1 (D) in the renal cortex of wild type and RenTg mice before and after irbesartan treatment. Note that while the expression of BMPs did not change, the expression of their endogenous antagonists noggin and USAG-1 was increased in RenTg and decreased to normal levels following treatment with the AT1 receptor antagonist. Values are mean±SEM; n = 5, 9 and 13 for wild type and RenTg mice before and after irbesartan, respectively; * P<0,05 or ** P<0,01 vs WT; ^#^ P<0,05 or ^##^ P<0,001 vs RenTg.

To get insights on the mechanisms accompanying the above-described structural and functional changes, measurement of gene expressions of pro- and anti-fibrotic genes of the TGFβ/BMP superfamily were performed (Figs. 8–9). Thus, expressions of the TGFβ itself, and of proteins acting either as co-factors (such as CTGF) and/or helping its action (such as Latent Transforming Growth Factor beta Binding Protein 4 or LTBP4) were several fold increased in 12 mo old RenTg mice (Fig. 8A–C). These differences in mRNA expressions were accompanied by the abnormal appearance of TGFβ within glomeruli and tubular epithelium in RenTg mice (Fig. 8E). AT1 receptor antagonism strongly inhibited this activation and reduced TGFβ, CTGF and LTBP4 expressions to normal levels (Fig. 8A–C & F). Irbesartan administration did not change expression of these genes in wild type mice.

Interestingly, the expression of proteins antagonizing the action of TGFβ such as BMP6 and BMP7 did not change (Fig. 9A&B) in 12 mo-old RenTg mice. However, the expression of agents acting as endogenous inhibitors/antagonists of BMPs such as noggin and uterine sensitization-associated gene-1 (USAG-1) were several fold increased in the renal cortex of 12 mo-old RenTg mice (Fig. 9C&D). Administration of the AT1 receptor antagonist decreased noggin and USAG-1 expressions back to normal levels (Fig. 9). Irbesartan administration had no effect on the expression of these genes in wild type mice.

## Discussion

In this study we investigated whether proteinuria and the associated structural and phenotype alterations can be reversed when therapy starts at advanced stages of renal disease, and we examined the mechanisms participating in this reversibility. An important novel finding is that podocyte lesions such as loss of foot processes and basal membrane disorganization are reversible phenomena when the local action of Ang II is blocked. An additional finding is that Ang II antagonism restores the normal phenotype of tubular cells and reverses renal vascular inflammation. Furthermore, we provide some clues about the mechanisms involved in the reversal of renal fibrosis: it appears that this recovery was due to a resetting of the TGFβ/BMPs equilibrium towards a more efficient action of the anti-fibrotic members of this family.

Regression of proteinuria and nephroangio-, glomerulo- or tubulointerstitial sclerosis by blocking the action of the renin-angiotensin system has been documented in several experimental models of progressive renal disease, such as chronic nitric oxide synthase inhibition, renal mass ablation, puromycin aminonucleoside [18]–[20]. However, a major scepticism about the reversibility of chronic kidney disease in rodents compared to humans, is that the disease was induced in most cases in young animals not suffering for a long period from a chronic disease like hypertension or diabetes (as is usually the case in humans), and that therapy was induced before reaching high levels proteinuria or an important destruction of podocyte structure for a prolonged period. To address these issues in the present study, we used a novel model [15], [16] of Ang II- induced renal disease mimicking closer the kinetics and the physiopathological characteristics of hypertension-associated human renal disease. These mice are hypertensive and display proteinuria (albuminuria) as early as 2–3 mo old, and these pathologies are accentuated with age and are accompanied by functional and structural alterations typical of chronic renal disease including loss of foot processes of podocytes and phenotype changes of renal epithelial cells towards the mesenchymal phenotype.

We decided to start treatment with an AT1 receptor antagonist when the animals reached the age of 12-mo, and thus, to treat relatively aged animals that had been proteinuric for a long period of their life-span. We found that treatment with an AT1 receptor antagonist induced reappearance of foot processes and de novo expression of nephrin and podocin (proteins indicating normal function of slit diaphragm) reducing thus, urinary protein (albumin) excretion. In agreement to our findings, downregulation of nephrin gene that was totally prevented by angiotensin-converting enzyme inhibitor and AT1 receptor blocker, was observed in a model of progressive renal disease (passive Heymann nephritis) [21]. In addition, Ang II induced redistribution of F-actin and zonula ocludents-1 (ZO-1) in a murine cell line of podocytes in vitro; these structural changes were mediated by the AT1 receptor and were associated to an increased permeability of albumin across podocyte monolayers. [22]. The repair of the glomerular function and the reversal of proteinuria can be due either to restructuring of existing and/or to de novo generation of podocytes. The first hypothesis, has been proposed for the reversal of glomerulosclerosis during ACE inhibition in the renal ablation model [23]. Alternatively, podocyte repopulation through remodelling of Bowman's capsule epithelial cells contributed to regression of renal disease in the Munich Wistar Frömter rat model of glomerular injury [24]. It is possible that the underlying mechanism of repair depends on the model and/or conditions of renal injury (abrupt hemodynamic changes in the renal ablation, much slower progressive decline in the MWF model). Our study is among the first using ultrastructural analysis and reporting re-appearance of foot processes and improvement of slit diaphragm barrier following therapy with AT1 receptor blockade.

In addition to glomerular, RenTg mice displayed also tubular epithelial lesions typical of chronic kidney disease. Thus, the expression of megalin, a tubular protein contributing to protein reabsorption and endocytosis was decreased in 12 mo-old RenTg mice, whereas it returned to normal levels following AT1 receptor antagonism. In agreement with an Ang II-induced effect on megalin expression, treatment with Ang II decreased mRNA and protein expression of megalin in a renal tubular cell line in vitro; this decreased expression resulted to a suppression of cellular uptake and degradation of albumin by these cells [25]. Decrease or loss of E-cadherin expression is a typical alarm signal indicating disappearance of the normal epithelial phenotype, whereas induction of FSP-1 expression is associated to the appearance of myofibroblasts and interstitial fibrosis. Thus, in addition to megalin decrease, RenTg mice showed decreased expression of E-cadherin concomitant to the appearance of tubulointerstitial fibrosis and increased expression of FSP-1; AT1 receptor antagonism reversed to normal these phenomena. Since numerous in vitro studies showed that E-cadherin loss and increased FSP-1 expression are induced by the action of TGFβ [26]–[28] and since TGFβ is considered as a major mediator of the pro-fibrotic action of Ang II [29]–[31], it is logical to propose that the changes in E-cadherin and FSP-1 expressions in the RenTg mice are due to the Ang II-induced activation of TGFβ pathway.

To address the above issue, we measured the expression levels of several members of the TGFβ superfamily. Comforting our hypothesis, expression levels of TGFβ, or of agents assisting or acting as co-factors such as CTGF and LTBP4 were upregulated in 12 mo old RenTg mice. Interestingly, the abnormal upregulation of TGFβ protein expression was evident in glomerular and tubular interstitial compartments of the kidney (Fig. 8E). This observation suggests that TGFβ was the common mediator of podocyte and tubular phenotype changes in RenTg mice. We have reported previously that Ang II induced collagen I synthesis and renal fibrosis through activation of TGFβ [9], [32]. In addition, systemic infusion of Ang II into normal rats increased renal CTGF mRNA and protein levels and induced renal injury; AT1 receptor antagonism prevented CTGF increase and the development of renal disease [33]. Other studies observed increased levels of CTGF associated to the development of proteinuria and interstitial fibrosis in murine models of diabetic nephropathy; in these studies, administration of CTGF antisense reduced proteinuria and serum creatinine and mesangial expansion [34]. Recent data strongly suggest LTBP-4 as an important regulator of TGFβ activation and the TGFβ-induced fibroblast differentiation associated to the development of fibrosis in tissues such as lung [35]. Our data suggest that a similar interaction between TGFβ and LTBP-4 also occurs during renal disease. The TGFβ superfamily includes the BMPs, which in addition to their bone morphogenic action are also antagonists of the pro-fibrotic action of TGFβ. In this regard, it has been shown that exogenous administration of pharmacological doses of BMP7 reversed renal disease in several models of experimental renal disease in rodents [36], [37]. In our model, the gene expression of major BMPs, such as BMP4 and BMP7 remained unchanged in the renal cortex of 12 mo-old RenTg mice before or after AT1 receptor antagonism. Of note, the expression of agents described as endogenous antagonists of BMPs such as noggin and USAG-1 strongly increased with the progression of renal disease in RenTg, and decreased to normal levels during AT1 receptor treatment. Recent studies showed that the USAG-1 is abundantly expressed in the kidney, and in kidney injury models, the ratio of USAG-1 to BMP-7 expression decreased with kidney damage but increased after subsequent kidney regeneration [38]. In addition, mice lacking USAG-1 are resistant to renal injury. Thus, USAG1-/- mice exhibited prolonged survival and preserved renal function in acute (cisplatin-induced nephrotoxicity) or chronic (unilateral ureteral obstruction) renal injury models. The preservation of the renal function in USAG-1-/- mice was attributed to the enhancement of endogenous BMP signaling and action [39]. Noggin is another agent antagonizing the BMP signalling pathway in a variety of cell and/or tissues in vitro [40], [41]. Very little is known about the Ang II effects on USAG-1 or noggin. To our knowledge, the present study is among the first reporting that the expression of endogenous antagonists of BMPs is induced during hypertensive chronic kidney disease and is reversed during AT1 receptor antagonism. It appears thus, that the development of fibrosis and chronic renal disease is not just a question of increased levels of TGFβ, but is a more complex process involving TGFβ, its co-factors, the BMPs and their endogenous antagonists, at least in the RenTg model. In this regard, it would be interesting to develop and test in renal diseases pharmacological agents specifically blocking the endogenous inhibitors of BMPs.

In conclusion, using a novel transgenic strain of mice we show that proteinuria and alterations in the expression of proteins involved in the integrity and function of podocytes or typical of the epithelial phenotype can be reversed when the local action of angiotensin II is blocked, even in aged animals with advanced renal injury. This recovery does not seem to result from correction in the phenotype of a particular cell type, but is rather the result of blocking the detrimental action of Ang II-TGFβ pathway in several types of renal cells simultaneously, leading thus to a functional and structural improvement in all renal compartments. Thus our study provides hope that patients suffering from chronic kidney disease would be efficiently treated and opens new insights on the mechanisms involved in this renal recovery.

## Materials and Methods

### Animals and experimental design

Experiments were performed using a transgenic strain of mouse (RenTg) backcrossed in the gentic background 129SV that is described elsewhere [15]. Briefly, RenTg mice express a renin transgene inserted into a liver-specific locus and driven by a liver-specific promoter/enhancer. The renin coding sequence (Ren2/1d) is a synthetic cDNA consisting of parts of the *Ren-2* and *Ren-1d* genes modified to include glycosylation sites for increased stability, a furin cleavage site to enable prorenin to active renin processing to occur in the liver and allow secretion of active renin into the blood stream. Thus, this transgenic strain expresses renin ectopically at a constant high level in the liver and leads to elevated mRNA and protein levels of prorenin and active renin.

The angiotensin II receptor antagonist irbesartan was administered to 12- month old male heterozygotes RenTg mice weighing 28–32 g at the dose of 10 mg/kg/day for a period of 6 weeks. Age-matched male wild-type mice were used as controls. A total of 22 RenTg and 10 wild type mice were used. All animals were handled in strict accordance with good animal practice as defined by the relevant national animal welfare bodies of France, and all animal work was approved by the appropriate committee of Inserm and the University Pierre et Marie Curie, Paris.

### Measurement of Systolic Arterial Pressure (SAP)

Systolic arterial pressure (mmHg) was measured with a tail-cuff sphygmomanometer adapted to the mouse, using automated system (MC 4000 BP analysis system, Hatteras instruments, Inc. Cary, NC). To avoid variations in blood pressure due to day cycle, all measurements were carried out between 9 and 11A.M. Animals were accustomed for several days before measurements. Only animals that did not display signals of stress and that showed stable and reproducible values of blood pressure for at least three consecutive days were considered for blood pressure measurements. 10 measurements from each mouse were taken at two minutes intervals then a mean value was determined.

### Measurement of urinary albumin excretion

All mice were acclimated in metabolic cages with free access to food and water for 24-hour urine collection. Measurements of microalbuminuria were performed using the Olympus System Reagent (OSR6167) and an Olympus AU 400 apparatus (Laboratory of Biochemistry IFR02, Paris). Urinary albumin concentration was normalized to urinary creatinine concentration, and values were expressed as g/mol Creat. Plasma samples were taken at the moment of sacrifice and creatininemia was determined by photometry.

### Renal morphology and Analysis of tubulointerstitial fibrosis

From each animal, one kidney was fixed in formalin solution (4%), embedded in paraffin after conventional processing, and sections (3 µm thick) were stained with Masson's trichrome for routine histological examination and Sirius red for detection of fibrillar collagen.

Interstitial fibrosis was assessed semi-quantitatively on both Masson's trichrome and Sirius red stained paraffin sections at magnification of ×20. Interstitial fibrosis was quantified using a computer-based morphometric analysis software (Analysis, Olympus) that allowed the formation of a binary image in which the stained area could be automatically calculated as percentage of the image area. Ten fields/specimen were randomly selected that covered nearly the whole piece of cortex. Scoring was performed blind on coded slides.

Immunostaining was performed on frozen (fixed in acetone, or in ethanol 70% or without fixation) and/or paraffin embedded sections. Paraffin sections were deparaffinized and rehydrated with baths of xylene and graded alcohol. Antigens were unmasked with citrate buffer (pH = 6 or pH = 9), proteinase K or pepsine 1X.

### Immunohistochemistry and Immunofluorescence (add antibodies for all proteins)

Three µm thick cryostat sections were placed onto super Frost^®^glass slides (Menzel GmbH & Co KG), and fixed with acetone for 3 min and stored at −20°C before use. Sections were treated with peroxidase for 10 min in order to block endogenous peroxidase activity. Then, sections were incubated 10 min with avidin and biotin. Between each incubation period, sections were washed in PBS. The antibodies used were anti-CD3 (1/100, Santa Cruz), anti-F4/80 (1/20, Abcam), anti-E-cadherin (1/100, Cell Signaling) and anti-megalin (1/250, gift from Dr. Verroust), anti-podocin (1/1000, gift from Dr Antignac), anti-TGFβ1 (1/100, Cell Signaling).

All steps were performed at room temperature. Rat and rabbit IgG were used as negative controls. Immunohistochemical quantification was performed using the Olympus analysis system described above and results were expressed as percentage of area.

### Total RNA extraction and quantitative Real time PCR

Total RNA was extracted from renal cortex using TRIzol reagent (Invitrogen) and methyl trichloride according to the manufacturer's instructions. RNA quality was checked by control of optical density at 260 and 280 nm, and by electrophoresis. Contaminating genomic DNA was removed by RNase-free DNAse (Quiagen) for 15 min at room temperature. cDNA was synthesized from 1 µg of purified RNA using oligodt and superscript II RT (Quiagen) for 1 h30 at 37°C and 10 min at 70°C. Real-time PCR amplification was performed with ABI PRISM 5700 Sequence Detection System using SYBR Green PCR Master Mix (Quiagen) as described previously [17].

Primers are listed in Table 1. All samples were assayed in triplicate, and the average value of the triplicate was used for quantification. Final results are expressed as the ratio of a given gene/gene reference (HPRT and/or 18 s) cDNA.

**Table 1:** List of the primers used for the Real Time-PCR of the different genes as mentioned in the results section.

Gene		Primers
Collagen Iα1	sens	GCAGGTTCACCTACTCTGTCCT
	antisens	CTTGCCCCATTCATTTGTCT
FSP-1	sens	GGAGCTGCCTAGCTTCCTG
	antisens	TCCTGGAAGTCAACTTCTTCATTG
Podocin	sens	CCATCTGGTTCTGCATAAAGG
	antisens	CCAGGACCTTTGGCTCTTC
Nephrin	sens	ACTACGCCCTCTTCAAATGCA
	antisens	TCGAGGGCCTCATACCTGAT
TGFβ1	sens	TGGAGCAACATGTGGAACTC
	antisens	GTCAGCAGCCGGTTACCA
LTBP4	sens	CCCAGCCCCATCGAGAAAG
	antisens	CAGTTGAGGGATACCTGACTCT
CTGF	sens	TGACCTGGAGGAAAACATTAAGA
	antisens	AGCCCTGTATGTCTTCACACTG
BMP-7	sens	CCTGGGCTTACAGCTCTCTG
	antisens	GGTGGCGTTCATGTAGGAGT
BMP-6	sens	TTCTTCAAGGTGAGCGAGGT
	antisens	TAGTTGGCAGCGTAGCCTTT
Noggin	sens	TGTGGTCACAGACCTTCTGC
	antisens	GTGAGGTGCACAGACTTGGA
USAG-1	sens	GCAACAGCACCCTGAATGAAG
	antisens	TGTATTTGGTGGACCGCAGTT
HPRT	sens	TCCTCCTCAGACCGCTTTT
	antisens	CCTGGTTCATCGCTAATC
18S	sens	GAGCGAAAGCATTTGCCAAG
	antisens	GGCATCGTTTATGGTCGGAA

### Urinary Excretion of MCP-1

Urine samples were conserved at −20°C and were centrifuged before measurements. Urinary MCP1 was measured by ELISA (Bender System), according to the manifacturer instructions. Each sample was assayed in duplicate.

### Transmission Electron Microscopy

Animals were perfused with 2.5% glutaraldehyde in 0.1 M sodium phosphate buffer at pH 7.4 (PB). Kidneys were removed, cut into small pieces, and immersed in 2.5% glutaraldehyde containing 1% tannic acid in 0.1 M PB for 2 h at 4°C. They were post-fixed with 1% OsO_4_, dehydrated and embedded in epoxy resin. Ultrathin sections were stained with uranyl acetate and lead citrate and then examined under a Philips CM10 electron microscope.

### Statistical analyses

Values are expressed as mean±SEM. Data were analyzed using one-way ANOVA followed by Protected Least Significance Difference Fisher's test of the Statview software package. Results with P<0.05 were considered statistically significant.
